# Improvement of Arm Movement in Volleyball Blocking Through Concurrent Augmented Tactile Feedback

**DOI:** 10.3390/sports14100454

**Published:** 2026-10-13

**Authors:** Aurelio Ureña, Enrique Ortega-Toro, Alicia Lara, M. Perla Moreno

**Affiliations:** 1 Department of Physical and Sports Education, Faculty of Sports Sciences, Sport and Health University Research Institute (iMUDS), University of Granada, 18011 Granada, Spain; aurena@ugr.es; 2 Faculty of Sports Sciences, University of Murcia, Sport Performance Analysis Association, 30107 Murcia, Spain; eortega@um.es; 3 Master in Physical Activity & Sport Research, Department of Physical and Sports Education, University of Granada, 18011 Granada, Spain; alichora@gmail.com

**Keywords:** representative learning design, Constraint-Led Approach, perceptual scaffolding, motor learning, sport initiation

## Abstract

The aim was to assess the usefulness of concurrent augmented tactile feedback delivered to the forearms for improving arm action during blocking. Twenty-three U-16 volleyball players (10 from a female team and 13 from a male team) completed a six-week intervention embedded in regular team practice. Training used a representative learning design with an additional over-net constraint providing concurrent tactile information to the forearms during blocking. Arm action changes were assessed using an ordinal observational scale, and players’ perceptions were explored through structured interviews. A cumulative mixed-effects ordinal model analyzed 570 blocking actions from 23 players. A significant overall improvement in arm action quality was observed from pretest to posttest (OR = 2.71; 95% CI [1.91, 3.86]; *p* < 0.001). However, a significant Moment × Team interaction was identified (*p* < 0.001). The female team showed a 3.38-fold greater increase in the odds of achieving a higher arm action score than the male team. Players’ perceptions of the practice were favorable according to the comments provided by the players during the interviews (84.62% female; 80.00% male), with participants highlighting a clearer understanding of the intended movement direction (30.77% female; 20.00% male). The findings support the value of tactile feedback as a training tool for the development of blocking skills.

## 1. Introduction

From an ecological perspective applied to sport, task, individual, and environmental constraints influence players’ movements and behaviors [1]. The Constraint-Led Approach (CLA) [2] emerges from this perspective and, based on the interaction between the individual and the environment, considers that the learner self-organizes to generate effective movement solutions through the interplay of various constraints [3]. Thus, manipulating constraints facilitates perception–action coupling and contributes to skill development through adaptation to a dynamic environment. According to Renshaw and Chow [4], the design of practical CLA environments should respect the following key principles: “(1) Constrain to Afford; (2) Representative Learning Design including (3) Purpose and Consequence and (4) Repetition without Repetition, which is framed around manipulating (5) Variability to increase or decrease (In)stability” (p. 111). Therefore, creating optimal learning environments where learners can search, explore, and exploit possibilities to achieve a goal should be a priority in task design [5].

Within the representative learning design approach, practice tasks should preserve the informational variables that are functionally relevant to competition and maintain meaningful perception–action couplings [6]. Representativeness does not require reproducing every feature of competition but rather preserving the functional relationships between information and action. Manipulating relevant perceptual information can alter movement organization, timing, and coordination, even when the nominal skill remains the same [7,8,9].

In sport, feedback and instructions have traditionally been provided verbally [10,11]. However, it may be beneficial to integrate multiple communication methods or to use verbal language to direct athletes’ attention toward other modalities of information delivery (e.g., visual or haptic) [11], that is, employing multimodal feedback [12]. This could make communication more effective and help address potential comprehension issues [13].

Furthermore, in the case of complex skills, when auditory or visual channels are overloaded, haptic feedback provides a more intuitive means of conveying information [14]. Conceptually, it is important to differentiate between the forms of somatosensory feedback involved in this process. Tactile feedback refers to the information received by cutaneous receptors in response to contact or pressure, which can guide the perception of limb position and movement [15]. Kinesthetic feedback, on the other hand, originates from proprioceptive receptors in the muscles and joints, providing information about position, motion, and tension [16]. The integration of both modalities gives rise to haptic experience, understood as the perceptual whole emerging from active interaction between the body and the environment [17].

The reviewed evidence indicates that augmented tactile information and haptic experience can modify motor performance [18,19], enhance specific somatosensory processes [20], become a functional source of action control through prolonged practice [21], and, under certain conditions, facilitate learning, retention, or transfer [22,23,24]. Studies have also identified potential limitations of haptic guidance, including the risk of dependency and difficulty maintaining the skill in an unassisted context [14].

**Figure 1:**
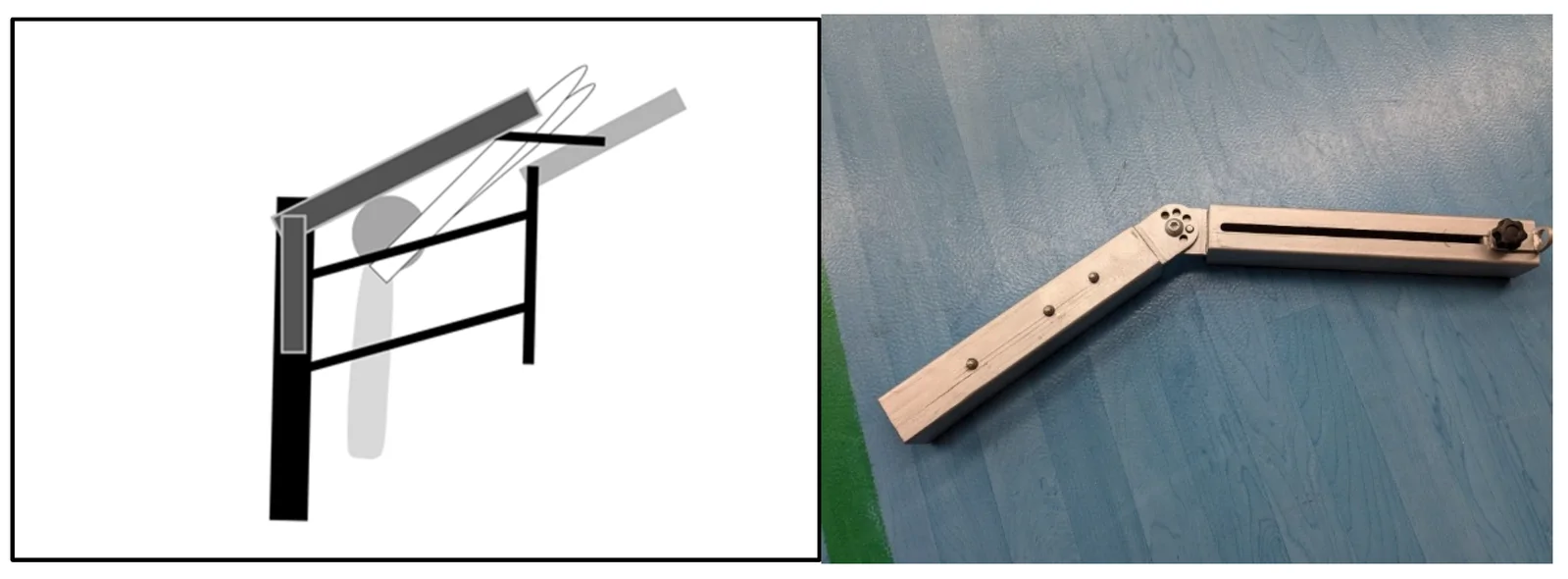
Diagram of the execution of the volleyball block using the “External Double-Height System for Net Sports” [28], as applied in volleyball, and photograph of the articulated arm of the “External Double-Height System for Net Sports.”

Recently, several studies have examined the characteristics of sports equipment used in various net sports and their potential for facilitating the manipulation of task constraints in different sports (e.g., tennis [25]; badminton [26]; volleyball [27]). Based on these works, a new piece of sports equipment, the “External Double-Height System for Net Sports” [28], has been developed. This device consists of a metallic implement attached to the net posts, allowing the placement of an elastic band at a chosen height above the net, acting as an auxiliary upper net. The implement is articulated, enabling the “over-net” to be tilted between 45° and 90° relative to the vertical plane of the net (Figure 1).

Blocking is one of the most decisive actions for team performance in volleyball, especially at high competitive levels. Studies on professional male teams have shown that successful blocking is highly correlated with final tournament rankings and is one of the main predictors of overall team performance [29]. In youth female categories, as the level of play increases, blocking gains greater tactical importance, although with higher technical error rates [30]. Lobietti [29] and Ureña et al. [31] consider blocking a complex, speed-dominated skill.

Arm action is a functionally relevant component of volleyball blocking because the movement and final position of the arms and hands determine the blocking surface presented to the attacker and its relationship to the plane of the net [32,33,34]. Experts have noted that dysfunctional movement patterns are common among young players [32].

Although evidence supports the contribution of constraints to movement modification and the value of representative practice tasks, a key challenge is determining how augmented information can be introduced to promote technical change without reducing task representativeness, and whether such change overcomes potential dependency or persistence problems when the constraint is removed.

From a perception–action coupling perspective, blocking is closely coupled to real-time affordances provided by the attacker, such as the trajectory of the set, the speed of the approach run, and the orientation of the hitter’s shoulder [35]. Therefore, the complexity, difficulty, and representativeness of blocking in match play should cause the tactile implement to function merely as a subordinate proprioceptive reference, secondary to the primary task objective of intercepting the opponent’s attack. If the device requires the blocker to overprocess each tactile stimulus, it may overload working memory and cause blocking technique to deteriorate under the pressure of actual play or fatigue [36].

Therefore, the aim of the present study was to assess the usefulness for improving arm movement during blocking of an implement that tilts the upper part of the net and provides concurrent augmented tactile feedback to the performers’ forearms. The intervention combined game-like situations in which the implement supported the blocking action, without any demonstration or explanation of arm movement, with game-like situations performed without concurrent augmented tactile feedback. To this end, the new “External Double-Height System for Net Sports” [28] was employed, providing concurrent augmented tactile feedback through contact between the players’ forearms and an elastic band extending over the net into the opponent’s court.

## 2. Materials and Methods

The study employed a one-group pretest–posttest design without a concurrent comparison condition. The absence of a comparison condition reflected the practical and logistical constraints of implementing the intervention within the teams’ regular training environment. Consequently, changes observed from pretest to posttest should be interpreted as associative and cannot be attributed exclusively to the intervention. The design also incorporated a qualitative analytical component.

The sample was selected through non-probabilistic convenience sampling and consisted of 23 U-16 volleyball players from two teams: one female team (*n* = 10; M = 15.5; SD = 0.52) and one male team (*n* = 13; M = 15.4; SD = 0.67). The teams competed at the local level and, within this U-16 context, displayed a low technical level of blocking and a limited contribution of blocking to performance, as this action requires a certain degree of game maturity and sufficient attacking ability. At this level and age, accumulated game experience with blocking is rudimentary; therefore, this period may be considered critical for refining the skill according to the technical criteria represented by the categories used for the study variable. Therefore, no additional information regarding players’ previous experience or playing position was considered necessary for the purposes of this study.

Informed consent was obtained from the parents or legal guardians of all participants in the study (Ethics Committee of the University, ID: 4733/2023).

The participation requirements were as follows: (a) belonging to one of the study teams; (b) having informed consent from the legal guardians; (c) attending at least 70% of the intervention sessions (8 sessions); (d) completing the interview used as a data collection instrument in the study. Specifically, players attended an average of 90% of the intervention sessions (range: 8–12 sessions) in the male team and 86% (range: 8–12 sessions) in the female team.

The intervention involved the use, within a volleyball training context, of a new piece of sports equipment, the “External Double-Height System for Net Sports” [28]. This device consists of a metallic implement attached to the volleyball net posts, allowing the placement of an elastic band at a chosen height above the net, functioning as an auxiliary over-net. The implement is articulated, enabling the over-net to be tilted between 45° and 90° relative to the vertical plane of the net (see Figure 1).

Using this implement in the court, with the over-net tilted, provided the players on the opposite side of the over-net with a spatial reference for the aerial invasion zone of the block into the opponent’s court, as well as tactile feedback when contact occurred with the blockers’ forearms.

The intervention was carried out over 12 training sessions, with a duration of 20 min per session. During that time, the “External Double-Height System for Net Sports” [28] was used with the elastic band positioned 10 cm above the official net height (2.18 m for the female category; 2.37 m for the male category) and tilted at 45° from the vertical. This setup caused the elastic band to extend 5 cm into the aerial space above one side of the court.

During each intervention period, a 6 vs. 6 exercise was performed, divided into games of 10 points. The serve remained with the team on the side where the implement extended 45° over the net into the opponent’s space, and after each game, both court sides and serving teams were switched.

At the beginning of the season, the team coaches agreed that, because a specific intervention would be implemented midway through the season, they would not previously provide instruction on blocking technique, except to direct players’ gaze toward the opposing attacker and encourage them to adjust to the attacker. Thus, until the intervention used in the present study was implemented, although the players had rudimentary experience performing blocks during play, they had received no specific instruction or information regarding arm movement during blocking.

The instructional protocol followed by the coach consisted of telling the players, during the warm-up phase in which the new equipment was used, at the beginning of the intervention, and whenever the coach considered appropriate during the intervention, that they should try to slide their forearm while maintaining contact with the band.

The coaches were instructed not to provide any prior explanation or demonstration of arm movement during blocking. They were also instructed not to provide verbal feedback about the movement or verbally reinforce successful contact with the band.

The intervention program was carried out over 12 training sessions conducted across six weeks, integrated into the two weekly training sessions of each team (the teams had a third weekly training session in which the intervention was not applied and which was left to the coach’s discretion, with the sole restrictions that blocking was not specifically trained and no feedback on blocking was provided). Each training session in which the intervention was applied lasted a total of 80 min. The structure of these sessions was as follows:

- Warm-up (30 min): Execution of the team’s usual warm-up routine, led by the coach. At the end of this phase, players familiarized themselves with the new sports equipment used in the intervention.
- Intervention (20 min): Use of the “External Double-Height System for Net Sports” [28] during 6 vs. 6 games, played to 10 points, switching court sides after each game.
- Real game (30 min): Standard 6 vs. 6 gameplays with the official net height and without the equipment used in the intervention.

The arm action during blocking was assessed at two specific moments: from the instant the hands rose above shoulder height until the opponent’s attack contact, and at the moment of the opponent’s attack contact. This variable was recorded using the criterion referred to as “arm action.”

The number of blocking actions was not predetermined for either assessment. Pretest and posttest were conducted using equivalent match-play formats and observation periods; however, because blocking opportunities emerged naturally during play, equivalent observation durations did not guarantee an equal number of blocking actions across assessment periods or players. All eligible blocking actions occurring during the recorded match play were included in the analysis.

In addition, players’ perceptions of the intervention were collected through a structured interview administered after completion of the intervention, within two days of the final intervention session.

Regarding arm action, technical descriptions identify forward batting of the arms as a common blocking error and emphasize that, during ascent, the blocker should keep the hands close to the net and seal the space between the top of the net and the arms ([32], p. 77). In addition, “batting on hitter contact might also create unnecessary net violations” ([32], p. 78). Likewise, “Arms actions (progressive action of sealing the net with the arms)” were identified by Palao et al. [27] as one of the criteria used to evaluate the quality of the technical blocking action. From a biomechanical perspective, effective blocking has also been characterized by the ability to position the hands above the net and penetrate its plane [33], with greater penetration contributing to increased court coverage and effective blocking area [34]. Therefore, arm action during blocking can be understood as a functional continuum, ranging from actions that fail to establish an effective blocking surface, through configurations that progressively close the space between the arms and the net, to projection of the upper limbs beyond the plane of the net.

**Figure 2:**
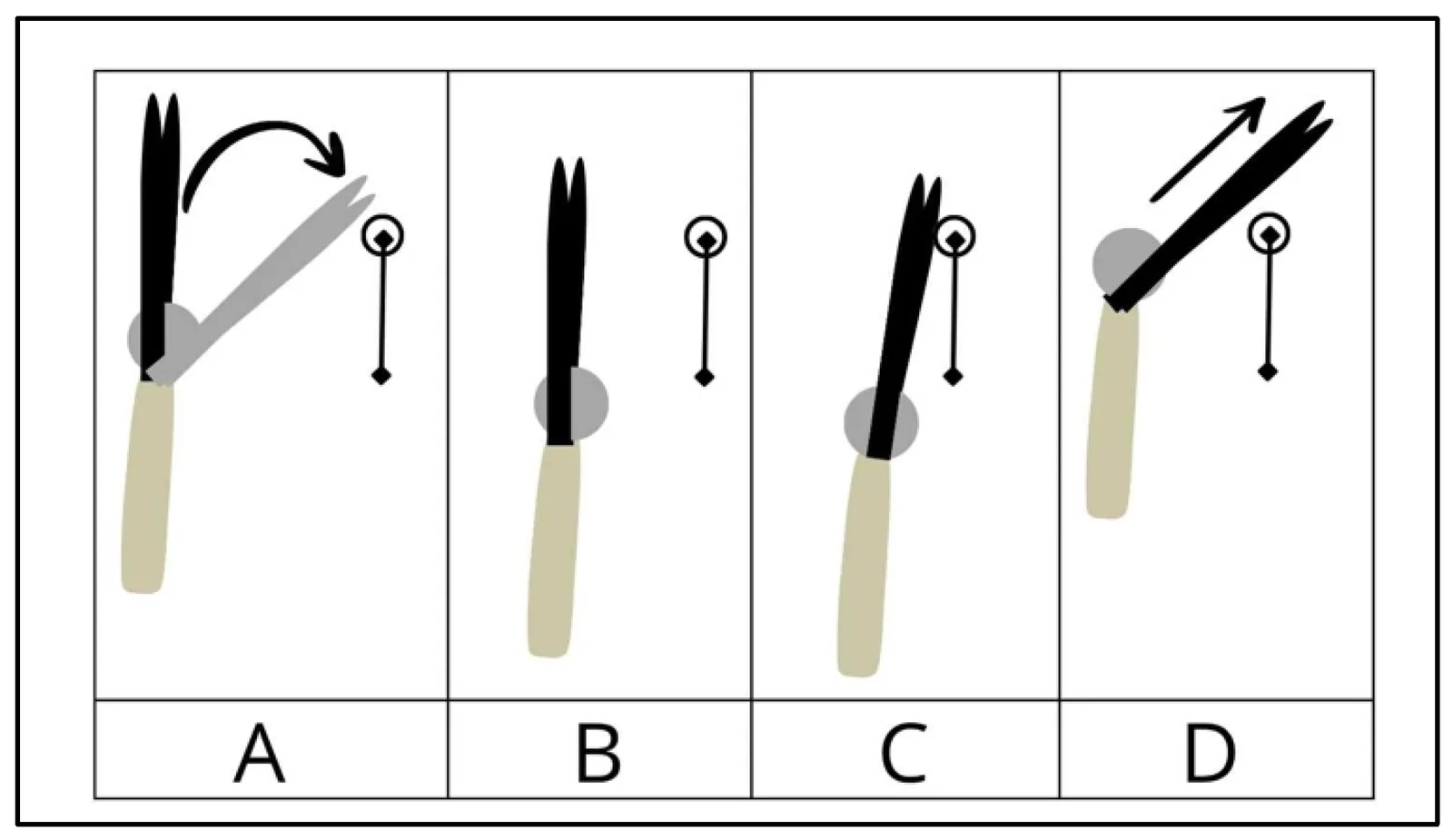
Schematic representation of the four observable types of arm action.

The arm action variable (Figure 2) was ordinal in nature, divided into four ordered categories ranging from least to most favorable:

A. Batting: The blocker’s arms, once elevated, perform a backward-to-forward movement characteristic of a striking action against the ball. (Value 0 on the ordinal scale).
B. Open: The blocker leaves a space equal to or greater than the diameter of a ball between their hands and the net. (Value 1 on the ordinal scale).
C. Closed: The blocker leaves a space smaller than the diameter of a ball between their hands and the net but does not cross the upper edge of the net into the opponent’s court. (Value 2 on the ordinal scale).
D. Penetration: The blocker’s wrists cross over the top of the net into the opponent’s court. (Value 3 on the ordinal scale).

The arm action variable in blocking was measured through systematic observation conducted by a specifically trained observer. Observer training comprised three sessions during which inter-rater agreement between the observer and two volleyball specialists acting as reference experts was assessed. The sessions were conducted at seven-day intervals. Agreement with the reference experts resulted in quadratic weighted Cohen’s kappa coefficients of 0.73 in the first session, 0.76 in the second session, and 0.85 in the third session. In each session, 20 blocking actions were observed, distributed across pretest and posttest recordings and male and female players, and selected at random. Observer training involved two separate coding decisions: first, identifying whether arm movement was posterior-to-anterior or upward (from the moment the hands rose above shoulder height until the opponent’s attack contact); and second, for upward movements, identifying the distance of the hands from the net (the opponent’s attack contact). During the observation process, blocking arm actions that raised doubts for the observer (11 actions) were reviewed and resolved by consensus between the observer and the two volleyball specialists who served as reference experts during training. One limitation of the observational procedure was that the video files were not presented to the observer under blinded conditions. Moreover, in the real-world context of an in-season team, it is very difficult to neutralize the elements that allow one session to be distinguished from another. However, the observer was unaware of the reference technical model and of the quality ranking and scores that would subsequently be assigned to each category of the variable. Furthermore, the observation procedure was divided into two separate coding decisions, which reduced the likelihood that the two behaviors would be integrated into a preconceived model of execution quality.

Recordings of the blocking actions during the three pretest matches and the three posttest matches were made using two Sony CX625 cameras, positioned laterally on both sides of the court (2 m from the sideline) and near the 3 m line closest to the net.

Players’ perceptions of the intervention were measured using responses to a structured interview consisting of two questions, administered after the completion of the intervention (within the two days following the final intervention session). The interview questions specifically addressed the perceived effect of the intervention on blocking and were as follows:

1. Do you think that training with the upper part of the net tilted toward the opponent’s court affected your blocking performance? If so, please specify how you think it affected you.
2. When you trained with the upper part of the net tilted toward the opponent’s court, during the blocking action, did feeling the contact of the elastic band on your forearms serve as a stimulus for you?

The interviewer was one of the researchers, was familiar with conducting interviews, had been trained for this purpose by another researcher with extensive experience in qualitative research, and held the highest volleyball coaching qualification (Level 3). The interviews were conducted at the same training facility, in a separate room near the playing court. After verbatim transcription of the interviews, a thematic analysis with a reflexive, inductive approach was conducted, following the guidelines of Charmaz [37] and Braun [38]. The process included: repeated comprehensive readings of the interviews; labeling of relevant data and subsequent thematic coding; identification of similar ideas to establish patterns; revision and determination of themes; naming and describing the themes; and linking the data to the players’ perceptions.

The unit of analysis used in the study was thematic, with a different unit identified each time a player referred to a distinct topic in their discourse. A “critical friends process” [39] was applied during transcription and coding, in which interpretations were discussed and refined collaboratively between the last author and the rest of the research team. This collaboration helped refine the thematic map by delineating subcategories, challenging the coder’s proposals, and consolidating specific and nonspecific subcategories, thereby ensuring a coherent and nuanced representation of the participants’ reported perceptions.

The study began with the signing of informed consent by the legal guardians of the participating players. Following this, the pretest was conducted, consisting of three training matches played to the best of three sets, carried out across three training sessions (one per week) among members of the same team.

The intervention, using the “External Double-Height System for Net Sports” [28], was then applied over a total of 12 training sessions, with a frequency of two sessions per week, resulting in an overall six-week intervention period.

Finally, players participated in the structured interview about their experience with the blocking intervention. Twenty days after the final intervention session (and following a two-week holiday period during which volleyball practice in training and competition was reduced), data were collected from the three posttest matches using the same format as that employed in the pretest.

Changes in arm action from pretest to posttest were examined using cumulative logit mixed-effects ordinal regression models. This approach was selected because arm action was assessed using four ordered categories representing progressively more favorable blocking actions (Batting, Open, Closed, and Penetration). Moreover, multiple blocking actions were recorded for each player across the two assessment periods; therefore, observations contributed by the same player could not be assumed to be independent. The player was consequently included as a random intercept to account for the clustering of repeated blocking actions within individuals and for between-player heterogeneity in the propensity to exhibit higher arm action categories.

Two complementary models were fitted. First, measurement occasion (Moment: pretest vs. posttest) was entered as a fixed effect to estimate the overall pretest-to-posttest change in arm action. Second, Moment, Team (female vs. male), and the Moment × Team interaction were entered as fixed effects to determine whether the magnitude of change differed between female and male team players. Both models used a multinomial probability distribution with a cumulative logit link function and included player as a random intercept.

Fixed effects were reported as regression coefficients (β), standard errors (SE), test statistics, odds ratios (ORs), and 95% confidence intervals (CIs). ORs for Moment were expressed as posttest relative to pretest; therefore, OR > 1 indicated greater odds of exhibiting a higher arm action category at posttest. The interaction was expressed as the female-to-male ratio of the posttest-to-pretest ORs. Following a significant Moment × Team interaction, simple effects of Moment were estimated separately for female and male teams as linear contrasts of the fitted model coefficients, with standard errors for derived contrasts calculated from the estimated parameter variance-covariance matrix.

Between-player heterogeneity was quantified from the variance of the player random intercept. To characterize the magnitude of within-player clustering, a latent scale intraclass correlation coefficient (ICC) was calculated as σ^2^ player/(σ^2^ player + π^2^/3), where π^2^/3 represents the residual variance of the standard logistic distribution. Statistical significance was set at *p* < 0.05.

The primary mixed-effects analyses were performed in IBM SPSS Statistics, version 30.0 (IBM Corp., Armonk, NY, USA) [40]. To verify the estimates and conduct model diagnostics, the cumulative link mixed models were independently reproduced in R version 4.5.1 [41] using the clmm function from the ordinal package (version 2026.7.26). The R models used a logit link, flexible thresholds, and adaptive Gauss–Hermite quadrature with 10 quadrature points. Model convergence was assessed from optimizer convergence status, the maximum absolute gradient, and positive definiteness of the Hessian. Numerical stability was additionally evaluated by refitting the models with 20 quadrature points and comparing the resulting fixed effect estimates.

Because the cumulative logit model assumes proportional odds, the constancy of the Moment and Moment × Team effects across the ordinal scale was examined diagnostically. Three cumulative binary mixed-effects logistic models were fitted, each retaining a random intercept for player, corresponding to the three possible cut points of the outcome: (1) Open/Closed/Penetration versus Batting; (2) Closed/Penetration versus Batting/Open; and (3) Penetration versus Batting/Open/Closed. These models were fitted in R [41] using the lme4 package (version 2.0.6). Variation in the estimated effects across these cut points was interpreted as evidence that the proportional odds assumption was not fully supported. Accordingly, threshold-specific models were retained as a supplementary robustness analysis to determine whether the substantive pretest-to-posttest pattern depended on imposing a common odds ratio across all category transitions. These analyses were based on the same 570 observations and accounted for clustering at the player level.

Because no a priori power analysis had been conducted, a simulation-based sensitivity analysis was additionally performed to determine the minimum effect magnitude detectable with approximately 80% power under the observed clustered design. Monte Carlo simulations reproduced the actual data structure (570 observations nested within 23 players), including the observed Player × Moment × Team configuration, and used the same cumulative logit mixed-effects framework as the primary analyses, with a random intercept for player. Model thresholds, nuisance fixed effects, and player-level random effect variance were calibrated from the fitted models, whereas the magnitude of the focal effect was systematically varied. For each simulated dataset, the corresponding mixed-effects model was re-estimated, and statistical detection of the focal effect was evaluated at α = 0.05 using likelihood ratio comparisons between models with and without that effect. Sensitivity was evaluated separately for the overall Moment effect and the Moment × Team interaction. A local search around the 80% power threshold was followed by 5000 Monte Carlo replications at the interpolated minimum detectable effect (MDE) for each focal effect. Simulations were conducted in R [41] using cumulative link mixed models fitted with the clmm function from the ordinal package.

## 3. Results

All 23 players contributed blocking actions at both assessments. The 323 pretest and 247 posttest observations reflect the number of blocking actions that occurred naturally during equivalent periods of match play rather than participant attrition or a predetermined number of observations per player. The female team contributed 256 actions (44.9%), of which 147 were at pretest and 109 at posttest, whereas the male team contributed 314 actions (55.1%), of which 176 and 138 were at pretest and posttest, respectively. In the female group, Batting decreased from 32.7% at pretest to 11.9% at posttest and Open from 29.9% to 14.7%, whereas Closed increased from 33.3% to 56.9% and Penetration from 4.1% to 16.5%. In the male group, Batting decreased from 3.4% to 1.4% and Open from 19.9% to 13.0%; Closed remained similar (54.0% vs. 54.3%), while Penetration increased from 22.7% to 31.2%.

The principal pretest-to-posttest effect remained statistically significant after accounting for the clustering of repeated observations within players (Table 1). The cumulative logit mixed-effects ordinal regression showed that the odds of exhibiting a higher arm action category were 2.71 times greater at posttest than at pretest (β = 0.998, SE = 0.179, OR = 2.71, 95% CI [1.91, 3.86], F(1, 566) = 31.20, *p* < 0.001). The player-level random intercept variance was significant, σ^2^ = 2.260, SE = 0.764, z = 2.96, *p* = 0.003, 95% CI [1.166, 4.383]. The corresponding latent scale ICC was 0.41, indicating substantial clustering of blocking actions within players and supporting the inclusion of player as a random effect.

The second model examined whether this pretest-to-posttest change differed between female and male teams (Table 1). The overall fixed effects model was significant, F(3, 564) = 16.93, *p* < 0.001. Moment was significant, F(1, 564) = 36.65, *p* < 0.001, as was Team, F(1, 564) = 7.84, *p* = 0.005, and the Moment × Team interaction, F(1, 564) = 11.71, *p* < 0.001. Given the significant interaction, group-specific pretest-to-posttest changes were considered the most informative interpretation of the model.

Simple effect analyses showed significant pretest-to-posttest changes in both groups, but with substantially different magnitudes. Among the female team, the odds of exhibiting a higher arm action category were 5.50 times greater at posttest than at pretest (β = 1.705, SE = 0.271, t(564) = 6.30, OR = 5.50, 95% CI [3.23, 9.36], *p* < 0.001). Among the male team, the odds were 1.63 times greater at posttest (β = 0.489, SE = 0.236, t(564) = 2.08, OR = 1.63, 95% CI [1.03, 2.59], *p* = 0.038). The Moment × Team interaction corresponded to an odds ratio of 3.38 (95% CI [1.68, 6.76], *p* < 0.001), indicating that the posttest-to-pretest increase in the odds of exhibiting a higher arm action category was approximately 3.4 times greater in the female than in the male group.

Significant between-player variability remained after accounting for Moment, Team, and their interaction, σ^2^ = 1.687, SE = 0.602, z = 2.80, *p* = 0.005, 95% CI [0.839, 3.396]. The corresponding latent scale ICC was 0.34, indicating that appreciable player-level clustering remained after inclusion of fixed effects.

The simulation-based sensitivity analysis indicated that, given the observed clustered data structure, approximately 80% power was achieved for an overall pretest-to-posttest effect of β = 0.485 (OR = 1.62; simulated power = 0.804) and for a Moment × Team interaction of β = 0.988 (ratio of ORs = 2.69; simulated power = 0.808). These values represent the approximate minimum detectable effects under the fitted ordinal mixed-effects models. The observed effects (OR = 2.71 for Moment and ratio of ORs = 3.38 for the Moment × Team interaction) exceeded these sensitivity thresholds.

The thematic analysis of the interviews showed that the majority of comments from the female players (84.62%) and the majority of comemments from the male players (80.00%) perceived that the applied intervention positively affected their blocking performance (Table 2).

Specifically, regarding how it affected blocking, 30.77% of the comments from female players and 20.00% from male players mentioned that they had a clearer movement direction when blocking. Examples of this are shown in the following excerpts (with fictitious player names):

- “Because my goal was to touch the elastic band.” (Anne)
- “Because I know exactly where my hands need to go.” (Hugh)

Players also perceived that they were able to penetrate more effectively during the block (23.08% of female comments; 20.00% of male comments):

- “Because now I put my hands further over the net to touch the band.” (Holly)
- “To put my hands further over, because before I held them straight up, and now with the band I’ve learned to direct them more toward the opponent.” (Lewis)

They reported blocking successfully on more occasions, i.e., having greater blocking success (23.08% of female comments; 6.67% of male comments):

- “My first successful blocks were thanks to that.” (Susan)
- “Blocking better as a middle blocker, covering all directions.” (Peter)

Players also mentioned that the intervention facilitated the execution of the block (20.00% of comments) and improved their concentration during blocking (13.33% of comments).

- “It’s a bit easier for me now.” (Hugh)
- “Positively, because I focus more on what I have to do.” (John)

Additionally, one female player (7.69%) mentioned the effect of trying to jump higher during the block.

Table 3 presents the thematic analysis of players’ perceptions of whether contact with the elastic band served as a stimulus. In this regard, it should be noted that Question 2 may have encouraged affirmative responses and therefore represents a potential source of bias.

The majority of comments indicated that feeling contact with the elastic band on the hands or forearms during blocking served as a stimulus (90.91% of female comments; 85.71% of male comments). Specifically, among the reasons given by female players for perceiving it as a stimulus were the assistance it provided (27.27%), as well as correct execution, court invasion, and concentration (9.09% each). Among the reasons specified by male players were correct execution and court invasion (28.57% each) and concentration, motivation, and assistance (7.14% each). The following excerpts illustrate these responses.

- “The band helps a lot when blocking because it helps you know where to position yourself and so on, so it is useful.” (Daisy).
- “I think so, because with the band you try to reach it with your fingertips and penetrate further into the opponent’s court.” (Chloe).
- “Yes, it has improved my blocking quite a lot.” (Jacob).
- “Yes, I was looking for it all the time, and it kept me more or less focused on the block instead of getting distracted.” (Archie).
- “Yes, it encouraged me, definitely.” (Luke).

## 4. Discussion

The main finding of this study was a pretest-to-posttest shift toward higher arm action scores during blocking. Across the full sample, the odds of exhibiting a higher arm action score were 2.71 times greater at posttest than at pretest. Lower level actions (Batting and Open) decreased, whereas Closed and Penetration actions increased. Importantly, the post-intervention assessment was conducted without the augmented over-net constraint and 20 days after the intervention had ended, indicating that the observed difference was not limited to performance while tactile information was available. The delayed, unassisted posttest therefore supports the persistence of the observed arm action change under unconstrained match-play conditions, but it does not, by itself, establish intervention-specific learning, transfer to other blocking skills, or improved blocking effectiveness. Because the study did not include a concurrent comparison condition, the observed pretest-to-posttest change cannot be attributed specifically to the intervention. Other time-varying influences, including regular practice, competitive experience, maturation, familiarization with the assessment context, and other changes occurring during the study period, cannot be ruled out.

The final position of the arms during blocking, particularly the height, orientation, and penetration of the hands above the net, is a key technical component that determines the ability to intercept attacks and reduce the opponent’s hitting angles [29,34]. Forward batting of the arms has been identified as a common blocking error in young players ([32] p. 77), whereas greater penetration contributes to increased court coverage and effective blocking area [33].

The threshold-specific analyses provide important nuance to the interpretation of the ordinal arm action outcome. The clearest and most consistent pretest-to-posttest change occurred at the transition from Batting/Open to Closed/Penetration. This distinction is particularly relevant in developmental players. Technical descriptions directed at formative stages identify forward batting of the arms as a blocking error and emphasize keeping the hands close to the net and progressively sealing the space between the arms and the top of the net [32]. Similarly, progressive sealing of the net with the arms has been proposed as a criterion of technical blocking quality [27]. Thus, progression from Batting/Open to Closed/Penetration appears to capture particularly well the arm action pattern targeted by the intervention.

Progression from Closed to Penetration requires a more cautious interpretation. Penetration beyond the plane of the net represents a biomechanically advantageous blocking configuration because it increases court coverage and the effective blocking area [33,34]. However, the evidence supporting this characteristic derives from adult players competing at high national levels. In developmental players, achieving this final configuration may depend not only on arm organization but also on the height reached above the net, and consequently on anthropometric and jumping constraints that were not targeted by the intervention. Therefore, the smaller effect observed at the Penetration threshold should not necessarily be interpreted as weaker change in the targeted arm action. This explanation remains hypothetical; however, anthropometric characteristics and blocking reach were not measured in the present study.

These technical and biomechanical sources provide the a priori rationale for identifying and ordering the arm action configurations; they do not constitute an empirical validation of the outcome against match-performance consequences within the present sample. The purpose of the present study was to examine whether observed arm action behavior changed in the direction represented by these established technical criteria, rather than to revalidate the criteria themselves. Accordingly, the outcome should be interpreted as a measure of arm action configuration rather than as a direct measure of overall blocking effectiveness.

The ecological paradigm posits that motor skill acquisition arises from the self-organization of coordinative behaviors in response to specific environmental, task, and individual constraints [1,2]. In this framework, “The practitioner, be it a Physical Education teacher or a coach, could be seen as a designer of practice” ([42], p. 2), manipulating task and environmental constraints to guide learners in discovering effective motor solutions.

A central concept here is the Constraint-Led Approach (CLA), which suggests that learning is optimized when environments restrict certain motor solutions and channel exploration toward functional patterns [3]. In this intervention, the introduction of a physical implement on the net providing tactile feedback on players’ arms acted as an environmental constraint, guiding execution without explicit technical instruction regarding the desired arm position or movement pattern. This tactile signal can be interpreted as a perceptual scaffold [42,43], which intentionally structures the practice environment to direct learners’ attention toward relevant information and promote exploration of functional movement solutions. The blocking device acted as a transient sensory support, facilitating perception of relevant information during early learning phases.

One of the main pedagogical challenges in sports training is to modify or simplify a task in order to guide technical learning without decontextualizing the skill, thereby avoiding the traditional analytical decomposition that disrupts the functional coupling between perception and action [6,44]. Empirical evidence indicates that removing informational variables representative of the actual performance environment, such as replacing a dynamic opponent with a ball machine or practicing without a real defender, substantially alters temporal coordination, kinematics, and movement organization [7,9]. Consistent with this issue, coaches should reduce the verbal prescription of technical solutions, which can overload athletes’ working memory with explicit rules and may foster dependence on concurrent feedback [36]. Instead, the coach should act as an environmental designer, selectively manipulating task constraints to guide and channel athletes’ proprioceptive and visual exploration within the search space [36,43,45].

Accordingly, the design omitted demonstrations and verbal information about technique and instead relied on concurrent tactile feedback, while emphasizing the representativeness of the blocking task within the game context.

In the post-intervention structured interviews, both female and male participants reported favorable perceptions of the practice, as reflected in 84.62% of the responses provided by female players and 80.00% of the responses provided by male players, which reported that the implement clarified the direction of movement “Because my goal was to touch the elastic” or “Because I know exactly where my hands need to go,” reflecting a positive perception of the usefulness of having a specific perceptual goal during learning. This supports Newell’s [1] concept of goal constraints within task constraints, which regulate action, direct perception and attention, motivate exploratory behavior, and establish foundational environmental relationships [46].

In addition, according to the players’ perceptions, the intrinsic relationship between the task goal and the feedback provided by the procedure acted as an incentive. Accordingly, most comments from female players (90.91%) and male players (85.71%) indicated that feeling contact with the elastic band on their hands or forearms while blocking served as a stimulus for improving the action.

Haptic feedback provides both kinesthetic information (body positions and movements) and tactile information (electro-tactile, thermal, or vibrotactile, depending on the actuator type) [47]. Marchal-Crespo et al. [48] developed haptic guidance as somatosensory feedback where a physical device applies adaptive forces to guide movement along desired trajectories, facilitating skill acquisition without high cognitive load. Given the clear functional differences between the physical device described above, functionally, the tactile implement in this study acted similarly as a perceptual scaffold, providing localized tactile feedback on the forearms during the final movement phase. Although it does not exert an active force that mechanically corrects the movement, accessory contact with the upper forearm acts as a somatosensory signal that may facilitate self-regulation of the movement, promoting a more efficient position without requiring instructions or demonstrations of the desired movement.

Approximately one-third of the residual latent variability was attributable to between-player differences rather than variation among repeated blocking actions within the same player. The player-level intraclass correlation coefficient (ICC) decreased from 0.41 in the overall model to 0.34 in the adjusted model. This indicates that technical behavior and its development cannot be fully characterized by group-level effects alone. Individual players appear to maintain different propensities to exhibit more advanced arm actions, underscoring the importance of considering individual trajectories and interindividual variability when evaluating technical interventions in blocking.

With recent technological advances, the use of this type of feedback has increased considerably in both sporting and non-sporting contexts. For example, visual–haptic feedback has produced significant improvements in body awareness and accuracy and reduced error rates, without imposing additional cognitive load, in ball interception skills performed in a virtual environment [49]. As Ren et al. [49] noted, ball sport training often lacks specific, personalized, real-time feedback that helps beginners develop rapid responses and precise movements, making haptic feedback or tactile information a promising alternative. However, because of the characteristics of these sports, many such studies have been conducted in laboratory or virtual reality settings rather than in actual game contexts.

In this study, although the augmented feedback was tactile, it occurred together with proprioceptive feedback, thereby forming a haptic experience that should be considered as a whole when evaluating the scope of the practice proposal. The findings suggest that this process may operate through mechanisms whereby the motor system adapts execution in response to sensory feedback, without following a prescribed movement pattern, potentially facilitating effective motor adjustments without interfering with voluntary control.

This study prioritized ecological validity, ensuring practice conditions resembled real-world application [9]. The intervention occurred during actual gameplay, with teammates and opponents, maintaining natural perceptual-motor demands. This aligns with ecological recommendations, as decontextualized practice may limit learning transfer [2]. In this case, the fact that the technical improvement occurred during 6 vs. 6 play under official rules provides a promising basis for hypothesizing possible adaptations to gameplay.

However, although numerous studies have supported the use of perceptual scaffolds and augmented feedback to promote adaptive motor learning [43,50] and have specifically shown that augmented tactile information and haptic experience can modify motor performance [18,19] and, under certain conditions, facilitate learning, retention, or transfer [22,23,24], other studies have identified potential limitations of haptic guidance. These include the risk of dependency or difficulty transferring the skill to unassisted contexts [14], the possibility that assistance produces only transient benefits [18,51], evidence that minimizing error does not necessarily maximize learning [24], and findings that adding haptic information does not guarantee superior transfer when other modalities, particularly vision, already provide sufficient information for task control [52].

The observed behavioral change is consistent with an interpretation based on the Constraints-Led Approach (CLA), operationalized through the provision of augmented tactile feedback. However, the present design does not allow determination of the magnitude of the influence exerted by the applied constraint, and therefore alternative theoretical mechanisms should also be considered. Predictive processing models provide one such explanation, according to which perceptual-motor adaptation may involve the updating of predictions through experience and prediction error [53].

According to this perspective, when players perform a particular arm action, they anticipate specific sensory consequences. The elastic band provides highly consistent additional information, visual and potentially proprioceptive/tactile depending on the nature of the interaction, regarding the relationship between the arms, hands, and the space above the net. When the expected action and the resulting sensory information do not match, prediction-error information may emerge and contribute to updating the underlying prediction. Through repeated experiences, the sensorimotor estimate associated with that action may be modified.

Furthermore, because the augmented constraint employed in this study was deliberately designed to channel arm action toward a technically defined solution, it is not possible to determine the extent to which the resulting behavior reflected exploration of the solution space as opposed to the prescriptive properties of the constraint itself [54]. The qualitative data revealed that a high proportion of player responses indicated a “clearer movement direction” (30.77% in females; 20.00% in males) and “greater concentration.” These subjective experiences suggest that the physical constraint did not operate in a purely biomechanical or unconscious manner but may also have influenced the athletes’ attentional focus and cognitive organization.

The Bayesian Integration framework complements this explanation by proposing that humans combine multiple sources of information, weighting them according to their relative reliability [53]. Players facing different organismic constraints or possessing kinematic priors associated with greater prior uncertainty may have assigned proportionally greater weight to the augmented tactile signal, thereby accelerating movement calibration [53]. This interpretation reinforces the recommendation proposed by Bobrownicki et al. [54] regarding the application of the coach’s Professional Judgment and Decision Making (PJDM), recognizing that the same constraint may generate heterogeneous adaptations depending on the athlete’s biopsychosocial profile.

Consequently, the use of an augmented tactile-feedback constraint may be viewed as a framework for guiding the design of the practice environment. However, the present study does not allow the underlying mechanisms responsible for the observed behavioral change to be determined.

Finally, regarding study limitations, the observer was not blinded to the measurement occasion. Although the observer was unaware of the reference technical model and of the quality ranking and scores subsequently assigned to each category, this lack of blinding may have affected the results. In addition, the sample was non-probability and convenience-based, comprising two specific teams. The absence of a concurrent comparison condition limits causal inference, and the changes observed between the pretest and posttest assessments cannot be attributed specifically to the intervention. Alternative time-varying explanations, such as regular practice, competitive experience, maturation, or familiarization with the assessment procedures, cannot be ruled out. Although the delayed posttest assessment, conducted without the additional constraint, documents the persistence of the observed arm action change over a 20-day period, no subsequent assessments were performed to determine how long this change persisted. Furthermore, the study design does not allow conclusions regarding broader transfer effects or improvements in blocking effectiveness.

In addition, the arm action categories were based on previously established technical and biomechanical descriptions from authoritative sources but were not validated against competitive performance outcomes within the present sample. Consequently, net violations were not recorded, and it is therefore not possible to determine whether the observed changes in arm action configuration were associated with changes in net-fault frequency.

Finally, because the male and female participants each belonged to a single team, team-specific characteristics cannot be reduced solely to sex differences. Therefore, sex cannot be interpreted as a definitive causal explanation for the differences observed between groups. The researchers chose to aggregate data from three match sessions for both the pretest and posttest assessments because blocking actions remain relatively infrequent in match play at these developmental ages.

The outcome measure was closely aligned with the technical behavior specifically targeted by the task constraint, and the observed changes should therefore not be interpreted as evidence of a generalized improvement in blocking skill. However, several features of the design distinguish the posttest from a direct assessment of compliance with the constraint.

As stated in the Conflict of Interest section, three of the four authors are co-inventors, together with researchers from other institutions, of the utility model used as research material in this study; although scientific and ethical standards were followed, this relationship may represent a limitation of the present study.

Several statistical and measurement limitations should be acknowledged. First, although the cumulative logit mixed-effects modeling approach appropriately accounted for repeated observations within players, threshold-specific diagnostic analyses indicated that the proportional odds assumption was not fully satisfied. Consequently, the common odds ratios derived from the cumulative-link models should be interpreted as summaries of a general upward shift in the ordinal distribution, rather than as evidence that the intervention exerted an identical effect across every transition between arm action categories. Accordingly, supplementary threshold-specific mixed-effects analyses were conducted to characterize this heterogeneity.

Second, the records from the three match sessions were not retained separately by date in the final dataset. Consequently, match-level clustering was not included in the models, and some residual dependence among observations recorded within the same match may therefore remain.

Finally, although Penetration represents the highest functional category within the arm action continuum, achieving this position in developmental players may depend not only on arm organization but also on anthropometric characteristics and blocking reach. Because these characteristics were not measured, it is not possible to determine from the present data the extent to which they contributed to the smaller magnitude of change observed at the Penetration level.

Since no a priori power analysis was conducted, the simulation-based sensitivity analysis partially addressed this limitation by quantifying the magnitude of effects that the observed clustered design was capable of detecting with approximately 80% statistical power.

## 5. Practical Applications

The approach examined in the present study appears to be most useful for athletes who are already willing and required to block but have not yet received specific information or instruction regarding arm movement technique. Because of the limitations inherent in conducting an experiment in a real-world context, practice in the present study was relatively concentrated. However, based on existing knowledge regarding practice distribution, a more distributed schedule for this type of session might provide additional benefits. External support should be withdrawn once the desired movement is regularly displayed during play. This creates a challenge because players within the same team differ in their rate of skill acquisition, requiring individualized solutions for those who progress more slowly.

## 6. Conclusions

The posttest showed a significant improvement in arm action during blocking, with a shift toward more favorable technical responses than those recorded at pretest. The findings suggest that the magnitude of technical change should be interpreted in relation to both interindividual variability and differences between groups with distinct initial distributions. These findings encourage further consideration of concurrent augmented tactile feedback as a support for technical development in contexts resembling competition during the developmental stages of blocking-skill acquisition.

The preservation of the teams’ usual training conditions in the present study limits the extent to which the net contribution of the intervention can be isolated. However, given the difficulty of executing the blocking skill at the developmental level examined and in light of the findings obtained, the present results provide a promising basis for future studies investigating the specific contribution of concurrent augmented tactile feedback to the development of blocking performance.

## Figures and Tables

**Table 1:** Cumulative logit mixed-effects ordinal regression models for arm action.

Model/Effect	Estimate	SE	OR	95% CI	Test	*p*
Overall pretest–posttest model						
Posttest vs. pretest	0.998	0.179	2.71	[1.91, 3.86]	F(1, 566) = 31.20	<0.001
Player random intercept	2.260	0.764	—	[1.166, 4.383]	z = 2.96	0.003
Moment × Team model						
Female: posttest vs. pretest	1.705	0.271	5.50	[3.23, 9.36]	t(564) = 6.30	<0.001
Male: posttest vs. pretest	0.489	0.236	1.63	[1.03, 2.59]	t(564) = 2.08	0.038
Moment × Team	1.216	0.355	3.38	[1.68, 6.76]	F(1, 564) = 11.71	<0.001
Player random intercept	1.687	0.602	—	[0.839, 3.396]	z = 2.80	0.005

Note. The dependent variable was ordered as Batting < Open < Closed < Penetration. Models used a multinomial distribution with a cumulative logit link and included player as a random intercept. For fixed effect rows, Estimate denotes β; for player random intercept rows, Estimate denotes σ^2^ and no OR is applicable. ORs for measurement occasion are expressed as posttest relative to pretest. The interaction OR is the female-to-male ratio of the posttest to pretest ORs. Latent scale ICCs were 0.41 for the overall model and 0.34 for the Moment × Team model. Omnibus fixed effect tests for the Moment × Team model were F(3, 564) = 16.93, *p* < 0.001 overall; Moment, F(1, 564) = 36.65, *p* < 0.001; Team, F(1, 564) = 7.84, *p* = 0.005; and Moment × Team, F(1, 564) = 11.71, *p* < 0.001.

**Table 2:** Descriptive analysis of players’ perceptions of the effect on blocking execution.

	Female (*N* = 13 Comments)		Male (*N* = 15 Comments)	
Categories/subcategories	Frequency	Percentage	Frequency	Percentage
Positive	11	84.62%	13	80.00%
- Clear movement direction	4	30.77%	3	20.00%
- Court Invasion	3	23.08%	3	20.00%
- Blocking Success	3	23.08%	1	6.67%
- Easier execution	0	0%	3	20.00%
- Concentration	0	0%	2	13.33%
- Jump	1	7.69%	0	0%
No perceived effect	2	15.38%	3	20.00%
- Unspecified	2	15.38%	3	20.00%

Note. *N* = 13 and *N* = 15 represent, respectively, the total number of comments analyzed for each team in this dimension, considering that each player could provide more than one comment. Percentages were calculated from these totals.

**Table 3:** Descriptive analysis of players’ perceptions of whether contact with the elastic band served as a stimulus.

	Female (*N* = 11 Comments)		Male (*N* = 14 Comments)	
Categories/subcategories	Frequency	Percentage	Frequency	Percentage
Served as a stimulus	10	90.91%	12	85.71%
- Correct execution	1	9.09%	4	28.57%
- Court Invasion	1	9.09%	4	28.57%
- Concentration	1	9.09%	1	7.14%
- Motivation	0	0.00%	1	7.14%
- Assistance	3	27.27%	1	7.14%
- Unspecified	4	36.36%	1	7.14%
Did not serve as a stimulus	1	9.09%	2	14.29%
- Unspecified	1	9.09%	2	14.29%

Note. *N* = 11 and *N* = 14 represent, respectively, the total number of comments analyzed for each team in this dimension, considering that each player could provide more than one comment. Percentages were calculated from these totals.

## Data Availability

Study data may be obtained directly from the corresponding author upon reasonable request.
